# The affective commitment of newcomers in hybrid work contexts: A study on enhancing and inhibiting factors and the mediating role of newcomer adjustment

**DOI:** 10.3389/fpsyg.2022.987976

**Published:** 2023-01-05

**Authors:** Alessandra Mazzei, Silvia Ravazzani, Alfonsa Butera, Sara Conti, Chiara Fisichella

**Affiliations:** Department of Business, Law, Economics and Consumer Behavior “Carlo A. Ricciardi”, Università IULM, Milan, Italy

**Keywords:** newcomers, onboarding, affective commitment, adjustment, hybrid work contexts, remote working

## Abstract

This study focuses on one of the most impacted human aspects of digital transformation in contemporary organizations: the development of the affective commitment of newcomers in hybrid work contexts. Specifically, this study addresses a research gap related to the factors that influence the affective commitment of newcomers in hybrid work contexts. First, it investigates the role of two drawbacks of the remote component of hybrid work contexts inhibiting affective commitment: workplace social isolation and technostress. Second, it explores the role of two factors that were previously investigated in in-presence contexts and proved to enhance affective commitment: perceived organizational support and perceived supervisor support. Moreover, this study considers the possible mediating role of newcomer adjustment, intended as a proximal outcome of successful onboarding and an antecedent of newcomer affective commitment. In order to examine enhancing and inhibiting factors and the mediating role of newcomer adjustment, a quantitative study was carried out involving newcomers who began to work in their current organization after January 2021 and who still do remote work at least 1 day a week. Results confirm the inhibiting role of workplace social isolation and the enhancing role of perceived organizational support and perceived supervisor support on affective commitment in hybrid work contexts. Furthermore, they support the mediating role of newcomer adjustment in the relationship between workplace social isolation and affective commitment. While contributing to theory advancement in understanding newcomer affective commitment in current hybrid work contexts, these results also suggest important managerial implications in the field of human resources management, specifically the need to pay greater attention to strategies devoted to increasing newcomers' perception of organizational and supervisor support.

## 1. Introduction

The human side of digital transformation in organizations has become increasingly relevant with the massive spread of remote working during the COVID-19 pandemic. Indeed, in recent years, remote working has become increasingly popular. Remote working “refers to the possibility of performing the working activity at a distance, maintaining the same working schedule and tasks, using suitable computer tools and portable personal computers connected to the corporate services” (Fregnan et al., 2022; p. 9). However, the progressive development of a new culture of work suddenly accelerated in 2020 due to the COVID-19 pandemic (Wevers, 2021). Suddenly, millions of workers were moved from the employer's premises to their own houses for a prolonged time to protect public and personal health as well as to assure business continuity. Although introduced or suddenly strengthened for emergency reasons, remote working for a high number of organizations and workers will continue to be in place in the future. Therefore, the future scenario will likely take the shape of hybrid work contexts (Gratton, 2021), where the employer's premises, employees' personal workstations, and digital platforms for collaboration will constitute a single work sphere (Kane et al., 2021; Wevers, 2021).

Hybrid work contexts present both benefits and drawbacks of remote and in-presence working. On the one side, the remote component of hybrid work contexts contributes to generating some benefits: an increase in productivity, autonomy, empowerment, and flexibility (Van Steenbergen et al., 2018); reduction in personal costs and time for commuting (Chung et al., 2020); better work–life balance (Kotera and Correa Vione, 2020); and increased employee satisfaction and reduced carbon footprint (Mortensen and Haas, 2021). The drawbacks of the remote component of hybrid work relate instead to risks of social isolation, workaholism, and technostress (Ipsen et al., 2020; Molino et al., 2020; Spagnoli et al., 2020; Zito et al., 2021); loss of colleagues' support (Kotera and Correa Vione, 2020); psychological burden of work–life blurring (Chung et al., 2020; Kotera and Correa Vione, 2020); and reduction in informal communication (Fay, 2011; Fay and Kline, 2011; Blanchard, 2021; Mortensen and Haas, 2021). On the other side, the in-presence component of hybrid work contexts has a series of strengths, namely smoother coordination, informal networking, stronger cultural socialization, organizational culture sharing, greater creativity, teamwork dynamics leading to innovation, and face-to-face collaboration (Fay, 2011; Kane et al., 2021; Mortensen and Haas, 2021). The drawbacks of the in-presence component of hybrid work can be seen as the inverse side of the advantages related to remote working.

In such working contexts, newcomer onboarding stands out as a process particularly challenged by the remote working component of hybrid working. Some of its drawbacks, such as technostress or the reduction in colleagues' support and informal communication, actually represent relevant challenges for newcomer onboarding in hybrid work contexts.

Specifically, newcomer onboarding is the process that supports newcomers' transition from being organizational outsiders to learning and adjusting to their new role and organization so as to become committed, engaged, and satisfied insiders who develop the intent of remaining with their organization (Feldman, 1981; Bauer et al., 2007; Saks et al., 2007; Saks and Gruman, 2011; Gruman and Saks, 2013; Perrot et al., 2014; Song et al., 2015; Chong et al., 2021; Vandenberghe et al., 2021). Research highlights the positive proximal and distal effects generated by newcomer onboarding: while adjustment to the role, organization, and organizational relationship network are considered proximal effects of effective onboarding (Bauer et al., 2007; Saks et al., 2007; Gruman and Saks, 2013), distal effects are related to newcomers' performance (Anakwe and Greenhaus, 1999; Bauer et al., 2007; Ellis et al., 2017; Chong et al., 2021), retention (Bauer et al., 2007; Saks and Gruman, 2011; Chong et al., 2021), and commitment to the organization (Bauer et al., 2007; Saks et al., 2007, 2011; Cooper-Thomas et al., 2020; Chong et al., 2021). Considering newcomers' commitment, in particular, Allen and Meyer (1990) referred to it as the affective attachment to an organization that involves shared values, a desire to continue to stay in the organization, and a willingness to exert effort on its behalf. In line with this conceptualization, this study considers the affective dimension of commitment that appears intrinsically connected to the willingness to perform well and to remain in the organization.

Building on the extant literature, Saks and Gruman (2012) explored the employee experience during organizational entry and identified what newcomers need the most, that is, *information* to reduce uncertainty and become aware of how to perform job tasks and adjust to the organizational life; *feedback* to reduce their entry anxiety to begin to perform at their best and to feel confident in themselves; and *social support* to deal with the pressures of a new job and related job demands.

In order to address the needs of newcomers and sustain their onboarding process, the organization can leverage a series of enhancing factors. For example, previous studies highlight *orientation programs*, i.e., employee training designed to introduce new employees to their job (Klein and Weaver, 2000; Saks and Gruman, 2012); *socialization tactics*, i.e., the ways in which the experiences of individuals who are adjusting to a new role and the organization are structured for them by the organization (Van Maanen and Schein, 1979; Jones, 1986; Ashforth et al., 2007); and *socialization agents*, who are organizational insiders such as supervisors and coworkers facilitating the adjustment to the new role and the organization by providing newcomers with information, feedback, resources, and support (Kammeyer-Mueller and Wanberg, 2003; Saks and Gruman, 2012; Vandenberghe et al., 2021).

The sooner newcomers feel welcome and prepared for their work, the sooner they can successfully contribute to the mission of the company. If joining an organization and a team when working in the office can be relatively quick, it gets more complicated when the process happens at a distance because of remote working (Cekuls, 2020). The process of newcomer onboarding and the consequent development by newcomers of relevant outcomes such as affective commitment to the organization can be inhibited by some of the drawbacks of hybrid work contexts that have been already highlighted, including reduced opportunities for informal and person-to-person contacts, increased stress due to the intensive use of technologies, and higher difficulties in sharing a common language and organizational culture. However, extant literature does not provide sufficient clues about the difficulties of the newcomer onboarding process in hybrid work contexts. Literature highlights a series of enhancing factors capable of sustaining newcomer onboarding, such as the support of socialization agents, the adoption of socialization tactics, and the implementation of orientation programs. Again, to date, extant literature has not clarified if and how those enhancing factors can sustain the newcomer onboarding process in hybrid work contexts.

This study contributes to filling the theoretical gap related to inhibiting and enhancing factors that can lead to the development of affective commitment to the organization in hybrid work contexts, where affective commitment is conceived as a crucial distal outcome of the newcomer onboarding process. This study, therefore, intends to advance theory and practice in relation to the key organizational process of newcomer onboarding in hybrid work contexts, contributing to a deeper understanding of the human side of digital transformation in contemporary organizations.

## 2. Literature review and hypotheses development

In order to shed light on the onboarding process through which newcomers are integrated into the organization and explore how they are impacted by the increased digitalization of the work context, this study proposes a research framework in which workplace social isolation, technostress, perceived organizational support, and perceived supervisor support are intended as antecedents that influence the development of affective commitment in hybrid work contexts, while newcomer adjustment is considered as a possible mediator in the relationship between each said antecedent and affective commitment. On this basis, a set of hypotheses is developed.

### 2.1. Affective commitment and the onboarding process

Onboarding is the process by which newcomers shift from being organizational outsiders to being insiders (Feldman, 1981; Bauer et al., 2007; Saks et al., 2007; Saks and Gruman, 2011; Gruman and Saks, 2013; Perrot et al., 2014; Song et al., 2015; Chong et al., 2021; Vandenberghe et al., 2021). Making newcomers feel part of the team and prepared for the job forthwith can contribute to a faster commitment to the organization (Bauer et al., 2007). Newcomer onboarding is the period when new employees start becoming familiar with new projects, procedures, and colleagues.

Through socialization tactics and socialization agents, the onboarding process leads the newcomer to adjust to the organization and the job, thus developing a series of *distal outcomes* in terms of *performance* and *job attitudes* (Fang et al., 2011). They include job satisfaction (Ashforth et al., 2007; Bauer et al., 2007; Cooper-Thomas et al., 2020), wellbeing (Cooper-Thomas et al., 2020), commitment (Ostroff and Kozlowski, 1992; Kammeyer-Mueller and Wanberg, 2003; Bauer et al., 2007; Saks et al., 2011), and intentions to remain (Cable and Pearson, 2001; Bauer et al., 2007; Saks et al., 2007; Cooper-Thomas et al., 2020). Such outcomes reflect unique and important attitudinal (commitment) and behavioral (work withdrawal and turnover) reactions to the workplace that have proved to be influenced by the degree of proximal learning and social integration on the part of the employee (Kammeyer-Mueller and Wanberg, 2003).

With specific reference to *affective commitment*, it is commonly conceptualized as an “affective attachment to an organization characterized by shared values, a desire to remain in the organization, and a willingness to exert effort on its behalf” (Allen and Meyer, 1990; p. 849). The affective dimension seems to be crucial: employees who display affective attachment are likely to develop a sense of belonging and identification that increases their involvement in the tasks, their disposition to pursue the organization's goals, and their desire to remain in the organization (Rhoades et al., 2001). In this sense, Rhoades et al. (2001) considered commitment as a discriminant of dedication and loyalty. Cohen and Veled-Hecht (2010) suggested that affective commitment is recognized as a stronger and more valid representative of organizational commitment in comparison to normative or continuance commitment, and for this reason, the authors maintained that affective commitment should be considered the key focus of organizational commitment.

Previous studies showed that effective onboarding increases the affective commitment of newcomers (Allen and Meyer, 1990; Cohen and Veled-Hecht, 2010). Institutionalized socialization tactics and structured experiences mitigate some entry anxiety of newcomers which, in turn, may enhance the attachment and identification with the organization and hence the development of affective commitment.

Thus considered, commitment is intended in its crucial affective dimension and is considered the key distal outcome of newcomer onboarding in this study.

### 2.2. Workplace social isolation

*Workplace social isolation* from colleagues and the workplace is a typical challenge of remote working (Buffer, 2020, 2021). It can be defined as a state of mind or self-belief to be out of touch with others (Golden et al., 2008), resulting from the self-perception of lack of support, missed opportunities for informal interactions (Toscano and Zappalà, 2020), and a lack of satisfying friendship relationships or access to social networks in the workplace (Marshall et al., 2007).

Extant literature detected three factors that are likely to influence the relationship between professional isolation and work outcomes (Golden et al., 2008): *the amount of time spent remotely*, since the higher it is, the greater the perception of professional isolation, given the fact that interactions are more likely to take place through devices that are less rich in sustaining relationships; *the extent of face-to-face interactions*, because these can reduce at least part of the communication and interpersonal obstacles, enabling contextual indicators such as head nods, gestures, and expressions, which facilitate quicker comprehension; and *the access to communication-enhancing technology* that facilitates connection and interaction with others.

Virtuality in teams can cause even more challenges compared to in-person settings: research found perceptions of isolation as one of the major issues in remote settings (Marshall et al., 2007). Specifically, the major problem related to isolation is the lack of frequent personal contact with team members, impacting the social support received (Orhan et al., 2016). Other challenges relate to employees' perceptions of a blurred social context and the loss of non-verbal cues during information transfer due to a lack of face-to-face interaction (Orhan et al., 2016).

Toscano and Zappalà (2020) also highlighted that the lack of face-to-face interaction with colleagues can be challenging in full remote working settings. This can be extended also to newcomers since social isolation inhibits relationship building, which is considered by scholars a relevant behavior that new employees should enact to favor their adjustment (Griffin et al., 2000). In this sense, Golden et al. (2008) highlighted that remote workers could be less confident in their abilities and knowledge, so they are at a position of disadvantage in performing their jobs. Problems regarding, for example, being less effective in managing interpersonal relationships or interactions with others can challenge the level of understanding derived from the sharing of tacit knowledge.

Marshall et al. (2007) highlighted that when virtual employees perceive the absence of supervisory help or direction, their perceptions of isolation likely increase, with a negative correlation between isolation and attitudes such as satisfaction and commitment. Communication and collaboration with coworkers and supervisors usually require intense face-to-face interactions, but remote working is an obstacle to this (Golden et al., 2008). Moreover, research studies found that newcomers are more likely to learn and internalize the key values of their new organization's culture if they have spent social time with socialization agents (Chatman, 1991; Cable and Pearson, 2001). Conversely, Wesson and Gogus (2005) showed that newcomers undergoing a computer-based orientation, compared to those taking part in face-to-face orientation, developed a lower understanding of the job and of the organization, in particular, regarding organizational goals, values, politics, and people.

Therefore, this study posits that:

*H1. In hybrid work contexts*, ***workplace social isolation*** *has a negative impact on the* ***affective commitment*** *of newcomers*.

### 2.3. Technostress

*Technostress* can be defined as “the stress that users experience as a result of application multitasking, constant connectivity, information overload, frequent system upgrades, and consequent uncertainty, continual relearning and consequent job-related insecurities, and technical problems associated with the organizational use of ICT” (Tarafdar et al., 2010; p. 304). Technostress arises from the extensive use of technology in one's job, the information overload, and the feeling of having to be always and everywhere connected and ready for a response (Tarafdar et al., 2010). It has been linked with the symptoms of anxiety and physical disorders, such as mental weakness, poor concentration, feeling of tiredness, and inability to sleep, impacting the personal and professional lives of employees negatively (Zito et al., 2021).

Technostress can be classified according to three dimensions: *techno-overload*, referred to situations where ICT obliges users to work faster and longer, modifying their work habits; *techno-invasion*, which regards the invasive effect of ICT on people's personal life, where employees feel the need to be constantly connected, making the boundaries between work and private contexts more blurred; *techno-complexity*, when the complexity of ICT makes employees feel inadequate concerning their skills (Tarafdar et al., 2010; Molino et al., 2020).

A research study found that the three technostress-related dimensions have been very impactful during the COVID-19 pandemic due to intense remote working, with relevant consequences on employees' burnout (Ninaus et al., 2021). In the current scenario that sees the progressive establishment of hybrid work contexts with part of employees continuing to work remotely, aspects such as the overload due to the use of technology as well as the difficulty in learning how to keep up with digital working tools represent stress creators for employees (Ninaus et al., 2021).

Research showed that technostress reduces individuals' job satisfaction and commitment to their organization (Ragu-Nathan et al., 2008). Considering newcomers more specifically, and the fact that their onboarding is a process aimed at reducing their anxiety and uncertainty in the transition to the new role and organization and ultimately leading to the development of their affective commitment to the organization, this study hypothesizes that in hybrid work contexts the additional source of stress represented by technostress can negatively influence the onboarding process, reducing the possibility for a newcomer to successfully develop an affective commitment to the organization.

Hence, this study posits that:

*H2. In hybrid work contexts*, ***technostress*** *has a negative impact on the* ***affective*** ***commitment*** *of newcomers*.

### 2.4. Perceived organizational support and perceived supervisor support

Scholars defined *perceived organizational support* as the extent to which individuals feel their organization cares about them (Eisenberger et al., 1986, 2002; Baranik et al., 2010). Literature evidenced the relationship between perceived organizational support and affective commitment (Rhoades et al., 2001), in the sense that when employees believe that the organization is committed to them, they feel to be committed to the organization (Baranik et al., 2010). Perceptions that the organization cares about employees who work for it are positively related to work attendance, job performance, citizenship behaviors, job satisfaction, and especially affective commitment to the organization. Specifically, a series of organizational HR practices seen as supportive by employees, and related to participation in decision-making, fairness of rewards and growth opportunities, increases perceived organizational support, and leads to affective commitment to the organization because of employee perceptions that the organization supports and cares about them (Allen et al., 2003).

Furthermore, perceived organizational support is considered to be able to generate greater affective attachment and feelings of obligation to the organization. According to the Organizational Support Theory, employees develop global beliefs concerning the extent to which the organization values their contributions and cares about their wellbeing (Eisenberger et al., 2002). However, employees must perceive the organization's commitment to them and the actions of organizational agents as discretionary in order to develop perceived organizational support (Eisenberger et al., 1986, 2002; Baranik et al., 2010).

With specific reference to newcomer onboarding, Allen and Shanock (2013) proved that socialization tactics influence perceived organizational support and job embeddedness, intended as the sense of being connected to the organization through a network of relationships. Their study also showed that perceived organizational support and job embeddedness are both related to affective commitment. Similarly, Simosi (2012) demonstrated that perceived organizational support has a direct effect on affective commitment. Through a meta-analysis of more than 70 studies on perceived organizational support, Rhoades and Eisenberger (2002) further confirmed that perceived organizational support is related to outcomes favorable to employees (e.g., job satisfaction) and the organization (e.g., affective commitment).

In addition, the literature highlights the specific relevance of *perceived supervisor support*. It is the perception of the beneficial treatment received from a supervisor that increases perceived organizational support to the extent that such treatment is attributed to the organization's policies, procedures, or general culture, and all this positively influences affective commitment to the organization (Rhoades et al., 2001; Simosi, 2012). In line with the definition of perceived organizational support, perceived supervisor support can be seen as the degree to which employees form impressions that their superiors care about their wellbeing, value their contributions, and are supportive (Eisenberger et al., 2002; Kurtessis et al., 2017).

Supervisors are in a unique position to support employees' competence needs by providing knowledge and feedback and to sustain their autonomy needs by directly influencing newcomers' work assignments and goals (Ashforth et al., 2007; Chong et al., 2021). Supervisors also have a strategic role in providing support and acting as role models (Anakwe and Greenhaus, 1999). Their actions also influence political knowledge, involving the informal network of power and interpersonal relationships in an organization and reducing the turnover hazard (Kammeyer-Mueller and Wanberg, 2003).

Supervisors make a major contribution to newcomer learning, given their formal authority to provide rewards, resources, work assignments, development opportunities, information, and feedback (Saks and Gruman, 2012).

During the period of onboarding, newcomers need *information, feedback*, and *social support* to reduce their feeling of uncertainty and anxiety and to manage the stress related to their new job (Saks and Gruman, 2012). In hybrid work contexts, the remote component of hybrid work challenges these expectations, since it induces risks of social isolation (Ipsen et al., 2020); loss of colleagues' support (Kotera and Correa Vione, 2020); and reduction in informal communication (Fay, 2011; Fay and Kline, 2011; Blanchard, 2021; Mortensen and Haas, 2021). Given these additional difficulties generated by remote working, perceived organizational support, on the one hand, and perceived supervisor support, on the other hand, may play a significant role in hybrid work contexts to develop the affective commitment of newcomers.

Hence, this study posits that:

*H3. In hybrid work contexts*, ***perceived organizational support*** *has a positive impact on the* ***affective commitment*** *of newcomers*.
*H4. In hybrid work contexts*, ***perceived supervisor support*** *has a positive impact on the* ***affective commitment*** *of newcomers*.

### 2.5. Newcomer adjustment as a mediator for affective commitment

The effectiveness of the onboarding process is related to the development of a series of proximal and distal outcomes. Literature (Kammeyer-Mueller and Wanberg, 2003; Bauer et al., 2007) showed that the proximal outcomes of the onboarding process are related to the adjustment of the newcomer to the organization and the job, in terms of the acquisition of knowledge, skills for the organizational role, and development of social relationships that help to bind the newcomer to the organization and its goals.

The most consolidated dimensions of newcomer adjustment are *role clarity* that consists of having sufficient information about the responsibilities and objectives of one's job in the broader organization as well as having knowledge of behaviors considered appropriate for achieving these goals (Rizzo et al., 1970; Feldman, 1981; Morrison, 1993; Kammeyer-Mueller and Wanberg, 2003; Bauer et al., 2007); *self-efficacy* or *task mastery*, i.e., the self-appraisal of one's ability to successfully fulfill job responsibilities (Morrison, 1993; Kammeyer-Mueller and Wanberg, 2003; Bauer et al., 2007); *acceptance by organizational insiders* or *social integration*, i.e., the perceived approval from coworkers and inclusion in their activities, which can be a source of social support and assistance (Morrison, 1993; Chao et al., 1994; Kammeyer-Mueller and Wanberg, 2003); and *knowledge of organizational culture*, i.e., the awareness of the organizational traits, values, goals, unwritten rules, internal politics, and language (Chao et al., 1994; Kammeyer-Mueller and Wanberg, 2003).

In their study on newcomer adjustment, Cooper-Thomas et al. (2020) proposed to converge the dimensions of adjustment into three domains: *role*, related to the understanding of the tasks the newcomer is responsible for, how tasks should be performed, and to what standard; *relationships*, related to the newcomer's need to establish effective and satisfying relationships with colleagues to become socially integrated; and *organization*, related to the understanding of the norms of the organization, including both formal aspects, such as its values, history, and structure, and informal aspects, such as the rituals and stories that illustrate how to behave and who wields power. The three domains of role, relationships, and organization capture the core content of socialization (Cooper-Thomas et al., 2020), and, in this study, they are considered the key components for newcomer adjustment.

Several authors (Kammeyer-Mueller and Wanberg, 2003; Bauer et al., 2007; Saks et al., 2007; Cooper-Thomas et al., 2020; Chong et al., 2021) posited that commitment, considered a distal outcome of the onboarding process, is associated with newcomer adjustment, intended as the proximal outcome of the same process.

Considering the demonstrated positive relationship between newcomer adjustment and affective commitment, this study hypothesizes that in hybrid work contexts, newcomer adjustment can play a mediating role in the relationship between workplace social isolation, technostress, perceived organizational support and perceived supervisor support, and affective commitment, respectively. Thus, this study posits that:

*H5. In hybrid work contexts*, ***newcomer adjustment*** *mediates the relationship between* ***workplace social isolation*** *and the* ***affective commitment*** *of newcomers*.
*H6. In hybrid work contexts*, ***newcomer adjustment*** *mediates the relationship between* ***technostress*** *and the* ***affective commitment*** *of newcomers*.
*H7. In hybrid work contexts*, ***newcomer adjustment*** *mediates the relationship between* ***perceived organizational support*** *and the* ***affective commitment*** *of newcomers*.
*H8. In hybrid work contexts*, ***newcomer adjustment*** *mediates the relationship between* ***perceived supervisor support*** *and the* ***affective commitment*** *of newcomers*.

**Figure 1:**
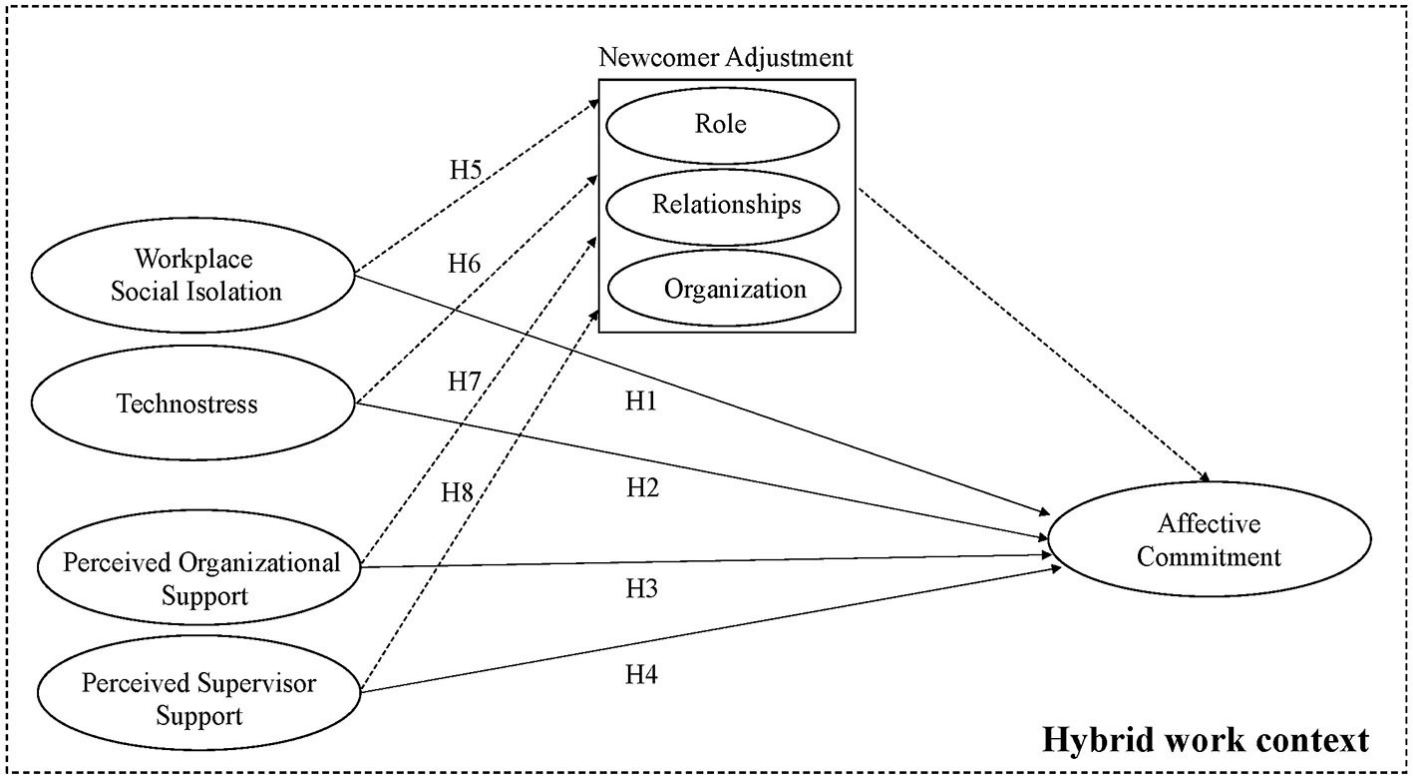
Research model.

According to the above hypotheses, this study's research framework is shown in Figure 1.

## 3. Methodology

### 3.1. Data collection

Data were collected through a web survey distributed by e-mail and social networks (Facebook and LinkedIn) between April and June 2022. Participants were newcomers who began to work in their current organization after January 2021 and who still do remote work at least 1 day a week. They were recruited through a convenience sampling technique and more precisely through the snowball method (Patton, 2002), based on the relationship networks of the researchers and their referral contacts. While this is a non-probabilistic technique that does not guarantee the representativeness of the population under study, it is especially useful when randomization is problematic because the population is large and unknown and still allows for detecting population members who are homogeneous according to the researchers' criteria (Etikan et al., 2015). The final sample included 109 participants that provided usable questionnaires. The questionnaire was in the Italian language to enable interviewees to clearly understand the questions and the research context. Brislin (1970) translation–back translation method was used for all scales described in the Measures subsection, except for the Technostress scale that was already available and validated in the Italian language by Molino et al. (2020).

### 3.2. Measures

The questionnaire included the following measures related to the variables considered in the research framework (see Figure 1).

*The affective commitment* was measured using the scale for affective commitment developed by Allen and Meyer (1990). The scale measures 8 items on a 5-point Likert scale from 1 (strongly disagree) to 5 (strongly agree). Examples of items are: “I would be very happy to spend the rest of my career with this organization” and “I really feel as if this organization's problems are my own.”

*Workplace social isolation* was measured using 7 items from the scale of Golden et al. (2008). Items were on a 5-point Likert scale from 1 (rarely) to 5 (most of the time). Examples of items are: “I feel left out on activities and meetings that could enhance my career” and “I miss out on opportunities to be mentored.”

*Technostress* was measured using the scale from Molino et al. (2020), which covers the dimensions of techno-overload, invasion, and complexity. Measures were on a 5-point Likert scale from 1 (does not apply at all) to 5 (applies fully). Four items explored techno-overload (e.g., “I am forced by technology to work much faster”); 3 items investigated techno-invasion (e.g., “I spend less time with my family due to technology”); and 4 items examined techno-complexity (e.g., “I do not know enough about technology to handle my job satisfactorily”).

*Perceived Organizational Support* was measured using the scale from Rhoades et al. (2001). It measures 8 items on a 5-point Likert scale from 1 (strongly disagree) to 5 (strongly agree). Examples of items are: “My organization cares about my opinions” and “My organization really cares about my wellbeing.”

*Perceived Supervisor Support* was measured using the scale from Rhoades et al. (2001). Four items from the perceived organizational support scale were adapted by replacing “organization” with “supervisor”. The items were on a 5-point Likert scale from 1 (strongly disagree) to 5 (strongly agree). Examples of items are: “My supervisor cares about my opinions” and “My work supervisor really cares about my wellbeing.”

*Newcomer adjustment* was measured using the scale from Cooper-Thomas et al. (2020) that covers the three domains of role, relationships, and organization. Responses were on a 5-point Likert scale from 1 (strongly disagree) to 5 (strongly agree), and each domain was measured with 5 items. An example of an item for each domain follows: “I understand how to perform the tasks that make up my job” (role factor), “I believe most of my coworkers like me” (relationship factor), and “I am familiar with the history of this organization” (organization factor).

### 3.3. Data analysis

The statistical software used for the analysis was IBM SPSS version 28.0 with the extension Macro PROCESS v.4.1. Frequencies, means, and standard deviations were calculated for all variables. Previously validated scales were used in this study. The reliability of all the scales was assessed using Cronbach's α. To evaluate possible effects of common method bias, Harman's single-factor test was conducted (Posdakoff et al., 2003) through confirmatory factor analysis: 35.7% of the variance is explained by the items of the six measures. This result showed that one single factor did not account for the variance in the data and suggested that common method bias was unlikely to pose a threat to the results of this study. The relationship between study variables was first explored by correlation analysis. A linear regression analysis was applied to verify H1–H4. Then, a mediation analysis was performed with Model 4 in PROCESS to test the mediation effect of newcomer adjustment (H5–H8). Bootstrap confidence intervals were used to determine whether the mediating effect was significant. The bootstrap resampling value was set at 5,000.

## 4. Results

### 4.1. Sample key characteristics and remote working conditions

In terms of gender, 65% of the sample was female population and 35% was male population. As regards age, 74% were up to 25 years old, 18% from 26 to 30 years old, and 8% from 31 years old onward. Considering education, 3% of respondents had a high-school diploma, 1% had a professional qualification, 20% had a bachelor's degree, 60% had a master's degree, 14% had a post-graduate specialization, and 2% had a Ph.D degree.

Considering the timing of when respondents began to work in their current organization, 11% started between January and March 2021, 8% between April and June 2021, 10% between July and September 2021, 17% between October and December 2021, and 54% between January and March 2022.

As regards the intensity of remote working, 19% of respondents worked remotely 1 day a week, 31% 2 days a week, 23% 3 days a week, 14% 4 days a week, and only 13% the whole week.

As far as the distance from their company's premises when working remotely is concerned, 50% of the respondents declared to be located at less than 10 km, 28% from 11 to 50 km, 12% from 51 to 100 km, and 10% more than 100 km.

Regarding the kind of job, 59% of respondents were doing an internship, whereas 41% were newly hired; 41% of respondents started their professional life in their current organization, whereas 59% were not at their first work experience.

Finally, 6% of respondents worked in companies operating in the manufacturing sector, 4% in public administration, 57% in companies operating in the service sector, and 33% in companies operating in other industries.

Table 1 summarizes the key characteristics of the sampled newcomers.

**Table 1:** Sample key characteristics and remote working condition.

**Characteristics**	**%**
**Gender**	
Female	65%
Male	35%
**Age**	
Up to 25	74%
From 26 to 30	18%
From 31 onward	8%
**Education**	
High school diploma	3%
Professional qualification	1%
Bachelor degree	20%
Master degree	60%
Post-graduate specialization	14%
PhD	2%
**Begin of the work**	
January–March 2021	11%
April–June 2021	8%
July–September 2021	10%
October–December 2021	17%
January–March 2022	54%
**Days of remote working in a week**	
1	19%
2	31%
3	23%
4	14%
5	13%
**Working situation**	
Internship	59%
Newly hired	41%
**First job**	
No	59%
Yes	41%
**Distance from the remote working site to the company site**	
Less than 10 km	50%
From 11 to 50 km	28%
From 51 to 100 km	12%
More than 100 km	10%
**Industrial sector of the employer company**	
Manufacturing	6%
Public administration	4%
Services	57%
Other industries	33%

Primary data. N = 109.

### 4.2. Correlation analysis

Table 2 presents the means, standard deviations, internal consistencies (Cronbach's α), and intercorrelations of the study variables. All reliability estimates were greater than 0.70, confirming the reliability of all scales in the Italian context too.

**Table 2:** Descriptive statistics and correlations of study variables.

	**Mean**	**Standard Deviation**	**Cronbach's α**	**1**	**2**	**3**	**4**	**5**
1. Newcomer adjustment	4.01	0.77	0.95	-				
2. Affective commitment	3.38	0.80	0.76	0.405^***^	-			
3. Perceived organizational support	3.75	0.85	0.90	0.515^***^	0.660^***^	-		
4. Perceived supervisor support	3.82	1.02	0.88	0.525^***^	0.599^***^	0.800^***^	-	
5. Workplace social isolation	2.44	0.95	0.88	−0.282^**^	−0.198^*^	−0.07	−0.214^*^	-
6. Technostress	1.93	0.69	0.88	−0.080	−0.053	−0.135	−0.161	0.343^***^

Primary data. N = 109,

^*^p < 0.05,

^**^p < 0.01,

^***^p < 0.001.

First, it is relevant to highlight that on average the sample shows a fair tendency toward newcomer adjustment (*M* = 4.01; *SD* = 0.77) and a downward tendency toward workplace social isolation (*M* = 2.44; *SD* = 0.95) and technostress (*M* = 1.93; *SD* = 0.69).

Second, nearly all variables result statistically significant and positively related to each other, except for workplace social isolation and technostress which are negatively correlated with the other variables but are significantly and positively correlated between themselves (*r* = 0.343, *p* < 0.001). Technostress has a weak and not significant correlation to other variables, except for workplace social isolation. Moreover, it is worth noting that workplace social isolation is not significantly correlated with perceived organizational support and that perceived organizational support is strongly correlated with perceived supervisor support (*r* = 0.800, *p* < 0.001).

### 4.3. Regression analysis

Table 3 reports the results of the regression analysis. In hybrid work contexts, workplace social isolation has a negative impact on affective commitment (β = −0.198, *p* < 0.05) and explains 4% of the variations in affective commitment (R-square value = 0.0394, *p* < 0.05). Thus, H1 is confirmed.

**Table 3:** Results of regression equations.

**Dependent**	**Variables**	**Unstandardized**		**Standardized**	***t*-value**	**Sign**.	**R-**	***F*-**	**Sign**.
**variable**		**coefficient**		**coefficient**			**square**	**value**	
		**B**	**Std**.	***B***					
			**error**						
Affective commitment (H1)	Costant	3.786	0.209		18.120	0.000	0.039	4.346	0.039
	Workplace social isolation	−0.166	0.080	−0.198	−2.085	0.039			
Affective commitment (H 2)	Costant	3.500	0.231		15.173	0.000	0.003	0.302	0.584
	Technostress	−0.062	0.113	−0.053	−0.550	0.584			
Affective commitment (H3)	Costant	1.043	0.263		3.958	0.000	0.436	82.791	< 0.001
	Perceived organizational support	0.623	0.068	0.660	9.099	0.000			
Affective commitment (H4)	Costant	1.579	0.241		6.552	0.000	0.359	59.863	< 0.001
	Perceived supervisor support	0.471	0.061	0.599	7.737	0.000			

Primary data.

H2 cannot be accepted instead: technostress does not have a significant impact on affective commitment in hybrid work contexts (β = −0.053, *p* > 0.05).

Finally, both perceived organizational support (β = 0.660, *p* < 0.001) and perceived supervisor support (β = 0.599, *p* < 0.001) have a positive impact on affective commitment and explain 43.6% (R-square value = 0.436, *p* < 0.001) and 35.9% (R-square value = 0.359, *p* < 0.001) of its variability, respectively. Therefore, both H3 and H4 are confirmed in hybrid work contexts.

**Figure 2:**
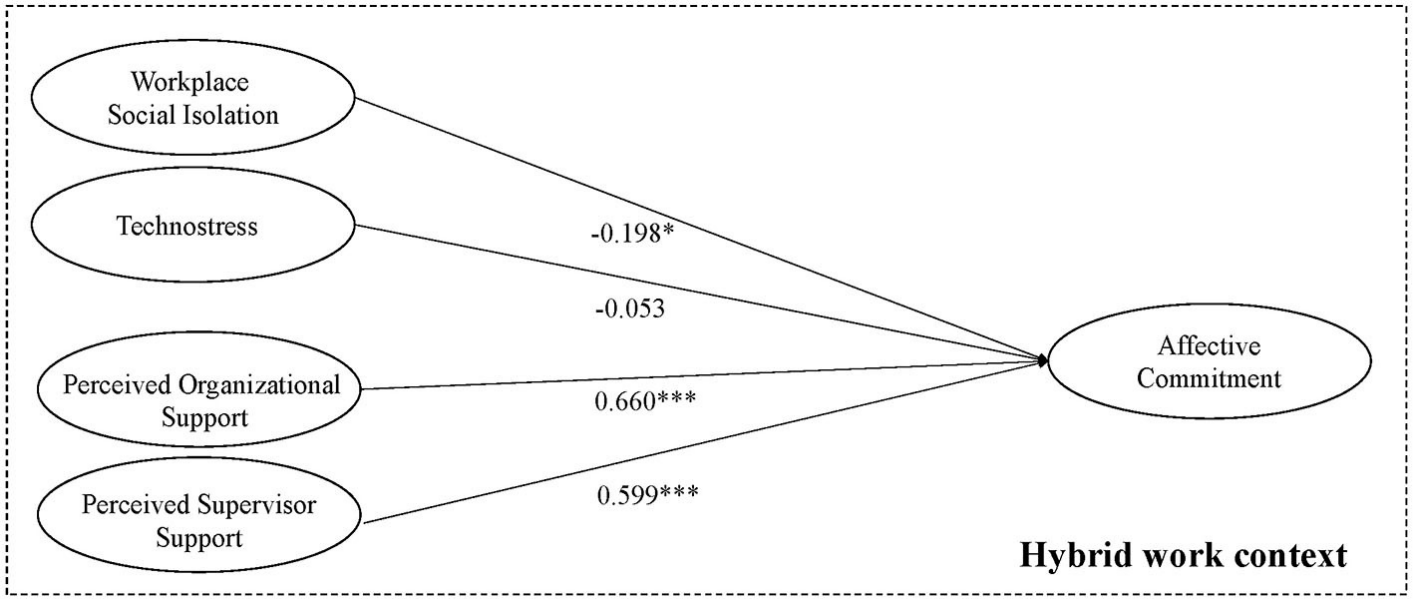
Research model with standard coefficients (H1–H4). Primary Data. *p < 0.05, **p < 0.01, ***p < 0.001.

Figure 2 visualizes the results of the regression analysis in the research model for H1–H4.

### 4.4. Mediation analysis

Model 4 of PROCESS was used to test the mediating effects. Total, direct, and indirect effects included in the model were described as statistically significant if the corresponding 95% confidence interval (CI) of the unstandardized effect size coefficient *b* did not contain zero. In valuing the significance of the indirect effect, a 95% bias-corrected CI based on 5,000 bootstrap samples was used.

**Figure 3:**
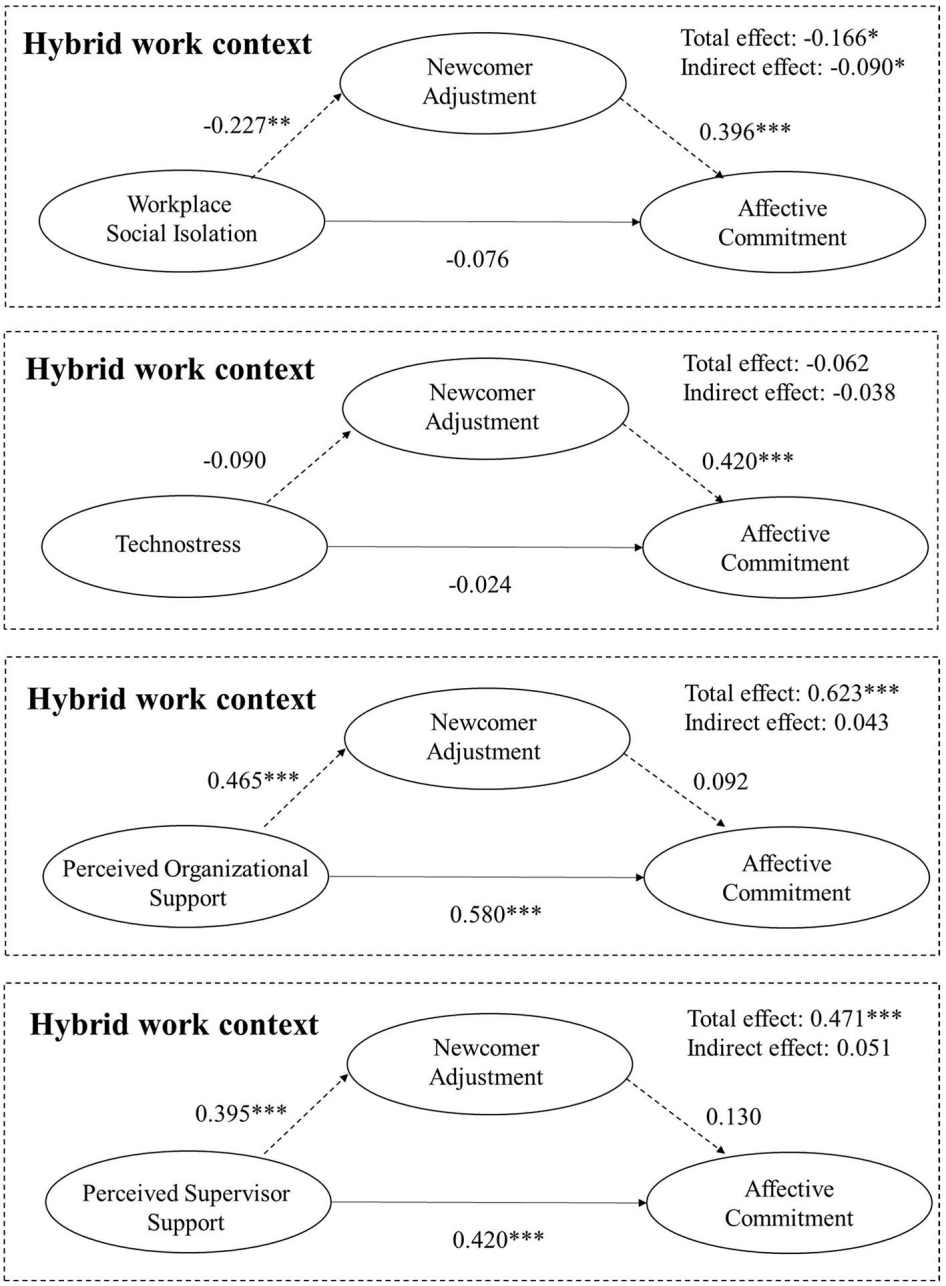
Research model with unstandardized coefficients (H5–H8). Primary data. *p < 0.05, **p < 0.01, ***p < 0.001.

Table 4 summarizes the results of the mediation models testing the effects of the four independent variables on affective commitment through newcomer adjustment. Figure 3 shows the unstandardized coefficients of the mediating models and their significance.

**Table 4:** Total (without mediator), direct (with mediator), and indirect (with mediator) effects.

**Variables**	**Type of effect on affective commitment**	**Effect**	**Std. error**	***t*-value**	***p*-value**	**95% CI**
Workplace social isolation	Total	−0.166	0.080	−2.085	0.039	[−0.3242; −0.0082]
	Direct	−0.076	0.077	−0.983	0.328	[−0.2299; 0.0775]
	Indirect	−0.090	0.048^*^			[−0.1999; −0.0139]^*^
Technostress	Total	−0.062	0.1126	−0.550	0.584	[−0.2850; 0.1613]
	Direct	−0.024	0.1039	−0.231	0.817	[−0.2300; 0.1819]
	Indirect	−0.038	0.050^*^			[−0.1548; 0.0423]^*^
Perceived organizational support	Total	0.623	0.068	9.099	0.000	[0.4874; 0.7590]
	Direct	0.580	0.080	7.267	0.000	[0.4220; 0.7386]
	Indirect	0.043	0.040^*^			[−0.0294; 0.1305]^*^
Perceived supervisor support	Total	0.471	0.061	7.737	0.000	[0.3503; 0.5917]
	Direct	0.420	0.071	5.888	0.000	[0.2783; 0.5608]
	Indirect	0.051	0.036^*^			[−0.0127; 0.1317]^*^

^*^Boot.

Primary data.

Findings reveal that workplace social isolation has a negative indirect effect (−0.090) on affective commitment through newcomer adjustment, where the bootstrapped 95% CI around the indirect effect (−0.1999; −0.0139) does not contain zero. Therefore, H5 is supported. The direct effect of workplace social isolation on affective commitment is not significant in the model. Consequently, newcomer adjustment totally mediates the relationship between workplace social isolation and affective commitment.

Technostress is confirmed as a not significant variable in predicting affective commitment even through newcomer adjustment. In fact, total (b = −0.062, *p* > 0.05), direct (b = −0.024, *p* >0.05), and indirect effects (0.038, bootstrapped CI = −0.1548, 0.0423) are not significant. Therefore, H6 cannot be confirmed.

Both perceived organizational support and perceived supervisor support have a significant total (respectively: b = 0.623, *p* < 0.001; b = 0.471, *p* < 0.001) and direct effect (respectively: b = 0.580, *p* < 0.001; b = 0.419, *p* < 0.001) on affective commitment. On the contrary, the indirect effect (respectively: 0.043; 0.051) on affective commitment through newcomer adjustment is not significant, where the bootstrapped 95% CI around the indirect effect (respectively: −0.0294, 0.1305; −0.0127, 0.1317) contains zero. Therefore, H7 and H8 cannot be confirmed.

## 5. Discussion

This study intended to clarify the role of enhancing and inhibiting factors on newcomer affective commitment in the newly emerging contexts of hybrid work, also considering newcomer adjustment as a possible mediator in the relationship between the identified antecedents and affective commitment. Deepening the understanding of newcomer onboarding is paramount. As highlighted by previous research, only when new organizational members become satisfied and engaged “insiders” and thus successful newcomer onboarding is realized (Saks et al., 2007; Saks and Gruman, 2011; Vandenberghe et al., 2021), such members develop a deeper commitment to the organization (Bauer et al., 2007; Saks et al., 2007, 2011; Cooper-Thomas et al., 2020; Chong et al., 2021). This has become even more critical in the face of the digital transformation in contemporary organizations accelerated by the COVID-19 pandemic.

The results of this study confirm that in hybrid work contexts affective commitment of newcomers is negatively related to workplace social isolation (H1) and positively related to both perceived organizational support (H3) and perceived supervisor support (H4). However, H2 related to technostress cannot be confirmed: findings point to a negative effect of technostress on affective commitment; however, this effect is not significant.

Workplace social isolation from colleagues and the workplace is a major challenge for remote workers, which causes negative effects such as a perception of lack of support and rewards and frequent social relationships at work (Marshall et al., 2007; Buffer, 2020, 2021; Toscano and Zappalà, 2020). The results of this study confirm the challenges that workplace social isolation, as experienced by newcomers in hybrid work environments, creates for their effective onboarding in terms of affective commitment development.

When looking at the findings of this study on perceived organizational support and perceived supervisor support, previous research has variously stressed that newcomers not only need information but also above all wide-ranging support to reduce their entry anxiety and deal effectively with the pressures of their new job and work context (Saks and Gruman, 2012). While job- and organization-related information can be provided, for example, through orientation programs and training, the perception of organizational and supervisor's support can be the demarcation line between successful and unsuccessful newcomer onboarding both in full in-presence work contexts and in hybrid work contexts. In fact, the results of this study validate the influence of perceived organizational support and perceived supervisor support as they make employees feel they are valued and supported and become affectively committed to the organization. Perceived organizational support has also been indicated by other scholars as essential for generating affective commitment (Rhoades and Eisenberger, 2002; Baranik et al., 2010; Simosi, 2012; Allen and Shanock, 2013). The fact that, in the current study, perceived organizational support resulted to be strongly correlated with perceived supervisor support may be explained by the fact that the latter contributes to the general feeling that the organization cares about and is committed to its employees. This is consistent with the results of previous research highlighting the essential role played by organizational agents and their discretionary actions for employees to develop a perception of organizational support (Eisenberger et al., 1986, 2002; Rhoades et al., 2001; Baranik et al., 2010; Simosi, 2012). Especially, in newcomer onboarding situations, supervisors have been described as critical socialization agents and role models (Kammeyer-Mueller and Wanberg, 2003; Saks and Gruman, 2012).

The contradictory finding related to technostress, which proved not to be significant to the development of the affective commitment of newcomers in hybrid work contexts, might be explained by the special conditions of employees entering new organizations during the COVID-19 pandemic: all of them had experienced a long and intense period of time studying or working online starting from 2020. Therefore, the prolonged learning process related to the use of technology could have mitigated the perception of overload, invasion, and complexity typically linked to the intense use of technology (Tarafdar et al., 2010; Molino et al., 2020). As a matter of fact, the entire sample shows a low level of perceived technostress (mean of 1.93 on a scale from 1 to 5).

Looking at the mediation models, newcomer adjustment, intended as the proximal outcome of the onboarding process in terms of knowledge developed by newcomers about their role and their organization as well as the effective relationships established with other organizational members, totally mediates the relationship between workplace social isolation and affective commitment. This suggests that the role of newcomer adjustment should be considered highly relevant in understanding and managing the dynamics of affective commitment development in hybrid work contexts where workplace social isolation can occur. However, contrary to the hypotheses of this study, it seems that newcomers can develop their affective commitment to the organization even if their adjustment is not completed, as long as they perceive to receive discretionary support by the organization overall and by their supervisors in particular. This could mean that when newcomers feel that the organization and their supervisors show concern about them, that their opinions, needs, and wellbeing are considered, and that their mistakes are understood, they start developing affective commitment even if not yet fully adjusted to the organization, i.e., they have not developed a full understanding of their role and the organization and they are not perfectly socially integrated.

## 6. Conclusion

This study focused on one of the most impacted human aspects of digital transformation in contemporary organizations: newcomer onboarding in hybrid work contexts. Specifically, it addressed the theoretical gap related to the factors that can influence the development of the affective commitment of newcomers in hybrid work contexts, where affective commitment is seen as a crucial distal outcome of an effective onboarding process.

This study provides several theoretical contributions. First, it highlights the inhibiting role of workplace social isolation on the affective commitment of newcomers also in hybrid work contexts. Second, it surprisingly reveals that the impact of technostress is not relevant for newcomer affective commitment in hybrid work contexts. It appears that the impact of technology is related to the learning process and its continuous use, contrary to expectations, reduces the perception of overload, invasion, and complexity.

Third, it shows that perceived organizational support and perceived supervisor support are two enhancing factors of the affective commitment of newcomers also in hybrid work contexts, while extant literature proved their effect in in-presence working contexts. The wide range of support given to newcomers to reduce their anxiety and the pressures of the new job and work context are still at the core of the onboarding process. While the perception of support received by the organization overall and by supervisors more directly sustains the development of the affective commitment of newcomers in hybrid work contexts, which in principle may be complicated by the drawbacks of remote working, such as social isolation, loss of colleagues' support, and reduced informal communication.

Fourth, it highlights that newcomer adjustment mediates the relationship between workplace social isolation and affective commitment, whereas it does not mediate the relationship between perceived organizational support and perceived supervisor support and affective commitment of newcomers in hybrid work contexts. It appears that in hybrid work contexts the perception of organizational and supervisor support sustains the development of affective commitment also in the case that the process of adjustment is not completed yet. This could mean that, although in hybrid work contexts the development of adjustment could be slowed down, newcomers can still start feeling affective commitment if they perceive organizational and supervisor support.

On the whole, this study contributes to the opening of a new avenue for the development of a specific model of affective commitment in hybrid work contexts.

Moreover, the results of this study lead to managerial implications and directions for newcomer onboarding practices in the “next normal” ahead (Sneader and Singhal, 2020). First, leveraging supervisors as role models and socialization agents is a key to increasing perceived organizational support. This can be facilitated by providing them with dedicated training and competence development paths to enhance their technological, relational, and dialogical skills.

Second, monitoring the possible effects of the frequency and duration of remote working should become common practice not to incur counterproductive effects, as well as investing in communication-enhancing technologies and in creating occasions for face-to-face interactions (Golden et al., 2008) also in the case of fully remote workers.

Third, organizations should consider revising the newcomer onboarding process in the face of hybrid work conditions, where differences in approaches, contents, and tools may be needed between those who work in-presence and those who partly or fully work remotely. For example, orientation programs could be designed so that newcomers can attend them on the company premises through face-to-face sessions instead of remotely through computer-based activities. This could help mitigate the negative effects of the remote component of hybrid working. Facilitating effective orientation and learning in hybrid work contexts seems particularly critical. Practitioners commonly refer to the 70:20:10 framework when developing learning and development programs where three types of learning are considered: experiential, social, and formal. While literature suggests overcoming some limits of this framework by planning and integrating all three aspects (Clardy, 2018; Johnson et al., 2018), in a hybrid work context, this effort of integration implies also the need for reducing the drawbacks of remote working when addressing the three components of learning.

This study has some limitations that future research could try to overcome. These are mainly associated with sampling. Future studies should enlarge the sample under study and possibly employ probability sampling techniques to increase the generalizability of the findings. For instance, the results of this study may have been biased by the high presence of female populations as well as of respondents doing an internship and thus having only a temporary contract. Future studies could also distinguish between the sectors to which newcomers belong. As this study sample mainly included workers from the service sector, it may be fruitful to analyze the onboarding process and its outcomes within those specific contexts in which hybrid work conditions are most likely to remain, such as Information Technology and Education. Moreover, this study could be replicated in the near future to see, from a longitudinal perspective, if any changes in the proposed research framework and studied relationships may occur due to the full realization of the work conditions for the “next normal” ahead.

Another suggestion for future studies is treating newcomer adjustment proximal outcomes (i.e., role, relationships, organization) as separate variables to weigh their relative contribution to and impact on affective commitment, as well as to assess their possible relative mediation to those antecedents of affective commitment whose impact was confirmed by the current study (i.e., workplace social isolation, perceived organizational support, and perceived supervisor support). Finally, the perception of support received by other socialization agents, namely, coworkers, could be studied in addition to the perceived support by the organization and supervisors.

In conclusion, the study's core contribution lies in improving the understanding of the impact of digital transformation on key organizational processes linked to the human side of organizations, and specifically of how remote working fueled by the COVID-19 pandemic has influenced the newcomer onboarding process. Specifically, this study has shown how workplace social isolation is an inhibiting factor that reduces the development of the affective commitment of newcomers in hybrid work contexts. At the same time, it highlighted, however, that the role of newcomer adjustment should be considered as a key mediator to fully understand the dynamics of affective commitment development in a situation of workplace social isolation that can occur in hybrid work contexts. On the contrary, the perception that newcomers have regarding the support received by their organization and by their supervisors can favor the development of the affective commitment of newcomers in such work contexts.

## Data availability statement

The raw data supporting the conclusions of this article will be made available by the authors, without undue reservation.

## Ethics statement

Ethical review and approval was not required for the study on human participants in accordance with the local legislation and institutional requirements. Written informed consent for participation was not required for this study in accordance with the national legislation and the institutional requirements.

## Author contributions

All authors listed have made a substantial, direct, and intellectual contribution to the work and approved it for publication.
